# Impact of Roux-en-Y gastric bypass on regulation of diabetes type 2 in morbidly obese patients

**DOI:** 10.1007/s00464-012-2160-4

**Published:** 2012-02-21

**Authors:** Monika Proczko-Markuszewska, Tomasz Stefaniak, Łukasz Kaska, Jarek Kobiela, Zbigniew Śledziński

**Affiliations:** Department of General, Endocrine and Transplant Surgery, Medical University of Gdańsk, Dębinki 7 Street, 80-952 Gdańsk, Poland

**Keywords:** Diabetes mellitus type 2, Metabolic surgery, Roux-en-Y gastric bypass, Incretins

## Abstract

**Background:**

The idea of surgery as treatment for type 2 diabetes mellitus (T2DM) was established in the US and was based on observation of patients after bariatric surgery. Resolution of T2DM is observed within a few weeks after surgery, in some cases even during hospitalization. The aim of this study was to evaluate the impact of Roux-en-Y gastric bypass (RYGB) on diabetes in morbidly obese patients.

**Methods:**

We present 73 patients with T2DM who underwent laparoscopic RYGB (LRYGB) to treat morbid obesity. In the group of 73 obese patients (mean BMI = 42.3), there were 41 females and 32 males.

**Results:**

Regression of T2DM was observed in 51 patients (69.8%) while hospitalized. In addition, 14 patients’ (19.1%) glycemia and HBA1c stabilized within 12 weeks after surgery (total regression rate of 88.9%).

**Conclusion:**

The ultimate evaluation of this method of treating T2DM is still lacking and requires several years of meticulous clinical studies. Despite that, considering the high cost of life-long conservative therapy of T2DM and its complications and the severe impact T2DM has on quality of life, surgical metabolic intervention may become the most reasonable solution in many cases.

Diabetes type 2 (T2DM) has been known as a pandemia or even a plague of the modern world. In 1995 there were 135 million people suffering from diabetes type 2 worldwide, while it is estimated for 2,030 that there will be more than 438 million with T2DM [1]. Diabetes is globally the fourth leading cause of death by disease. T2DM has become the most frequent condition in people with kidney failure in Western countries. It is also estimated that more than 2.5 million people worldwide are affected with diabetic retinopathy. People with T2DM are more than twice as likely to have a heart attack or stroke as nondiabetic people. T2DM is also one of the most frequent complications of obesity, diagnosed in more than 20% of obese patients. On average, people with T2DM live 5–10 fewer years than nondiabetics [2].

T2DM is considered a social disease and has a significant impact on health-care funding. Those expenses include both direct (diagnostics, treatment, and hospitalizations) and indirect costs (sick leaves, early retirement, home care of patients). In the US the annual cumulated cost of treatment of T2DM patients reaches 100 billion dollars [3].

This has led to an intense search for some alternatives to the current treatment of T2DM and its consequences. At the moment one of the most interesting alternatives is surgical treatment of T2DM. The idea is based on the observation of the postoperative course of bariatric patients. Several studies have documented remission of T2DM in 47–70% of cases after restrictive procedures, 80–98% after RYGB, and a rate as high as 92–100% after biliopancreatic diversion (BPD) [4].

The first attempts of surgical intervention in T2DM therapy were undertaken between 2004 and 2006. Rubino et al. [5, 6] published a series of experiments on the antidiabetic properties of metabolic procedures. They found that T2DM resolved within 3 weeks after surgery, with an average glycemia level of 96.3 ± 10.1 mg/dl.

It is well known that surgical procedures originally designed to treat morbid obesity cause remission or significant improvement of T2DM. The metabolic mechanisms of hyperglycemia control still remains elusive, but there are some data suggesting that mechanisms independent of losing weight may play an important role in the surgical treatment of diabetes.

The role of gastrointestinal surgery in T2DM resolution was included in a consensus of 53 experts on diabetes who met in Rome in 2007 (1st International Conference on Gastrointestinal Surgery to Treat Type-II Diabetes, Clinical and Research Guidelines Development, Rome 2007, Italy). In that document surgical treatment was considered acceptable for patients with BMI = 30–35 in whom T2DM is difficult to control in a standard conservative way. The next step was made in February 2010 during the Diabetes Surgery University of Malaga meeting when it was concluded that metabolic surgery procedures could be performed in all patients 18–65 years old with insulin-dependent, difficult-to-control T2DM, as well as in patients treated with oral hypoglycemic drugs who are considered to be transferred to insulin therapy [7, 8].

In March 2011 in New York at the 2nd Congress on Interventional Therapies for T2DM [9], a multidisciplinary team of clinicians, scientists, and policy makers discussed the role gastrointestinal surgery in eligible patients and developed an agenda of research priorities to set a new consensus on the role of surgical treatment of T2DM in a group of obese and nonobese patients.

The aim of this study was to evaluate the impact of Roux-en-Y gastric bypass (RYGB) on diabetes in morbidly obese patients.

## Material and methods

Our prospective study included 73 patients with T2DM who underwent laparoscopic RYGB (LRYGB) to treat morbid obesity (BMI > 35 kg/m^2^, mean = 42.3) between 2008 and 2010. There were 41 women and 32 men (M:F ratio = 0.78) with a mean age of 51.1 ± 11.3 years (range = 37–62). LRYGB was chosen for all patients because of its potential as a concomitant curative procedure for T2DM. The preoperative period lasts 3–4 months and depends on cooperation with the dietician, the psychologist, and the bariatric surgeon. Usually during this time there are three meetings with each of these specialists. The nonsurgically achieved weight loss did not improve glycemia significantly. The average duration of T2DM was 8.2 ± 4.7 years.

The American Diabetes Association (ADA) 2009 Standards of Medical Care in Diabetes defines T2DM as fasting (8 h) plasma glucose (FPG) ≥ 126 mg/dl [10]. The National Institutes of Health (NIH) guidelines defines normal FPG as ≤99 mg/dl [11]. The ADA glycemic goal for glycosylated hemoglobin (HbA1c) for nonpregnant adults is <7%. The ADA document refers to this range as “a nondiabetic range of 4.0–6.0% using a DCCT-based assay” [12].

In all cases, the preoperative HbA1c and the highest and lowest blood fasting glucose level were determined. In addition, the dose and type of antidiabetic agents (insulin or oral hypoglycemic agents) and the duration of the disease were taken into account. Similarly, the HbA1c was assessed 4 weeks after surgery and then every 3 months, and glucose levels were measured on postoperative days 0–4 every 4 h until discharge from hospital. The levels of glucose were then observed for 16 weeks and were presented as a daily average of all measurements. The reports of the need for diabetes medications after surgery were also analyzed.

All the patients were qualified for LRYGB. Intestinal anastomosis was performed after exclusion of biliopancreatic limb (80–130 cm) and digestive limb (100–140 cm).

Routinely, in all cases an intraoperative methylene blue test was performed. On postoperative day 4 an X-ray of upper GI tract with water-soluble contrast was taken to exclude anastomotic leak. Postoperative follow-up took place according to the following schedule: the first visit was 4 weeks after surgery, followed by visits at 3, 6, 12, and 18 months and every year for the next 5 years.

## Results

In a group of 73 patients, regression of T2DM was observed in 51 patients (69.8%) while still hospitalized. In addition, 14 patients’ (19.1%) glycemia and HBA1c stabilized within 12 weeks after surgery, which makes a total regression rate of 88.9%. The criteria for diagnosing resolution of T2DM included the level of HbA1c <6% and glucose fasting level <100 mg/dl. In the remaining 8 (11%) cases with difficult-to-control T2DM, there was still need for antidiabetic medication, but glycemic control was much more effective.

**Fig. 1:**
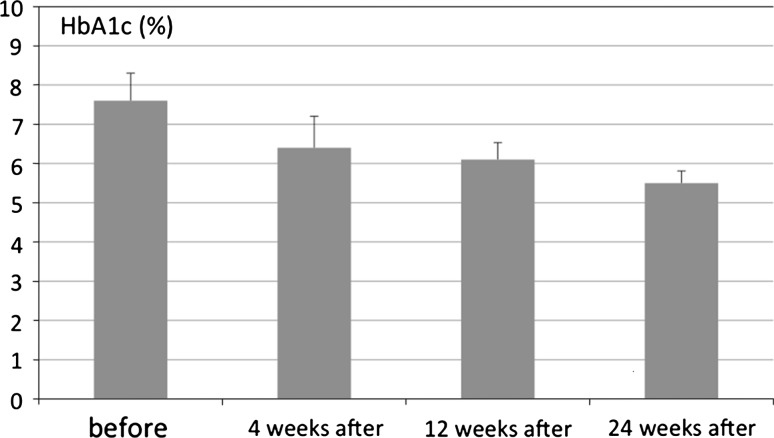
Level of HbA1c before and after laparoscopic Roux-en-Y gastric bypass

**Fig. 2:**
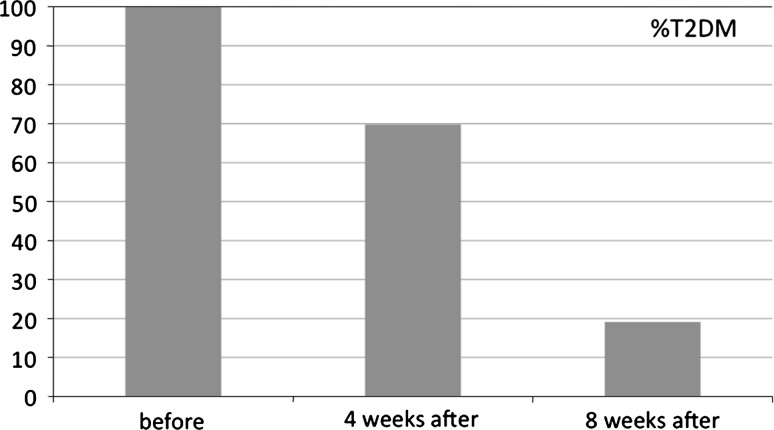
Postoperative resolution of type 2 diabetes mellitus

The average difference between the highest and the lowest measurements of blood glucose (Δ) 4 weeks after LRYGB was 68.3 ± 10.4 mg/dl. The average HbA1c 4 weeks after surgery was 6.4 ± 0.8%, and 8 weeks after LRYGB it was 6.1 ± 0.43% Figs. 1 and 2; Table 1.

**Table 1:** Results of LRYGB in group of patients with T2DM and BMI > 35 kg/m^2^

		HbA1c (%)				T2DM resolution % of cases	
HbA1c (%) before RYGB	∆ mg/dl (highest – lowest glycemia level)	4 weeks after RYGB	12 weeks after RYGB	24 weeks after RYGB	∆ mg/dl 4 weeks after RYGB	4 weeks after RYGB	8 weeks after RYGB
7. 6 ± 0.7	190 ± 13.2	6.4 ± 0.8	6.1 ± 0.43	5.5 ± 0.31	68.3 ± 10.4	69.8%	19.1

We have also noticed better glycemia regulation and faster T2DM resolution in the group of 73 patients with BMI > 35 kg/m^2^ in whom the excluded biliopancreatic limb was longer than 100 cm compared to those in whom this excluded limb was shorter than 100 cm. In the group of 51 patients in whom the resolution of T2DM was observed just after surgery during the hospital stay, 30 had a biliopancreatic limb length of 100–130 cm (*p* = 0.0345). In the group of 14 pathologically obese diabetics in whom glycemia was stabilized in the period of 12 weeks after surgery, 5 had excluded biliopancreatic limb length over 100 cm (*p* = 0.0692). In the eight cases that did not have resolution of T2DM but did have improvement of glycemia control, the excluded biliopancreatic limb was shorter than 100 cm; in only one case it was 120 cm.

## Discussion

The main reason behind the incidence and progression of T2DM is increasing peripheral insulin resistance together with impaired endocrine function of pancreatic β cells [13]. This dysfunction results in the lack of ability to produce insulin in response to changes of glycemia. Despite oral hypoglycemic therapy or even insulin therapy, further progression of β-cell insufficiency is being observed. It is suspected that the background of this pathology is associated with defect of the intestinal hormones, incretins, which are suspected to play an important role in regulation of the metabolism of glucose. Incretins act in various pathways. First, they increase the production of insulin by β cells and suppress secretion of glucagon. Second, they slow down stomach emptying and suppress appetite. They also are supposed to increase peripheral insulin susceptibility.

Pathological incretin reaction in T2DM constitutes mostly decreased secretion of glucagon-like peptide 1 (GLP-1) with maintained insulinotropic effect [14, 15]. At the same time, secretion of gastric inhibitory peptide (GIP) is within physiologic values but its effectiveness is severely impaired, which results in inadequate transportation and utilization of glucose in peripheral tissues. It has been noted that after RYGB the secretion of GIP decreases in patients with T2DM, but this has not been seen in obese but nondiabetic patients [16].

It is the effect of incretins that has been perceived as the most important mechanism behind the miraculous resolution of diabetes after RYGB unrelated to the weight loss. Apart from that, it has also been hypothesized that an unknown biochemical signal/hormone can be produced in the duodenum. This signal/hormone would be responsible for resistance of the peripheral tissues to insulin. It has also been speculated that one or more unknown substances called anti-incretins, possibly blocking insulin activity, can be produced in the initial part of small intestine [17, 18].

Duodenal bypass and surgical connection of the stomach with the medial part of the jejunum results in shortening the period of absorption of nutrients, which directly leads to the weight loss. The results of surgical treatment of obesity have been proven to be successful in reducing body weight, but it is also successful in the resolution of concomitant diseases and alleviating the risks of vascular diseases and cancer [19, 20].

The analysis of the results of surgical treatment of obese patients with T2DM has also proved unquestionable effectiveness: normalization of glycemia has been observed in most of the patients within a few weeks postoperatively, and in some of them as early as during hospitalization [21].

Rubino et al. [22] emphasized the role of gastrointestinal tract endocrine mechanisms and underlined that exclusion of the duodenum from the nutrient pathway was more important than faster transportation of nutrients to the terminal part of ileum, where incretins are produced.

The results of a 20-year Swedish Obese Subjects (SOS) study indicate an important success of bariatric surgery, i.e., reduction of the risk of heart attack and stroke in patients with diabetes. Researchers compared 2,010 patients after bariatric surgery with 2,037 nonsurgical patients who received medical treatment or lifestyle modification for morbid obesity. The study found that in obese patients with T2DM, a striking 70% remission was observed 2 years after bariatric surgery. At 15 years after surgery, 30% remained in remission [23]. It is worth noting that the surgical procedure described in that study was mostly gastric banding which, as merely a restrictive procedure, does not employ hypothetical incretin mechanism and, therefore, is not as effective in T2DM treatment in comparison to RYGB.

Currently, several randomized trials are under way comparing surgery with medical therapy in adults with diabetes and BMI <35. The results of those studies will help to formalize recommendations for patients with BMI of 30–35, who form the second-largest group of patients with diabetes [24]. Encouraging results have been presented by Schauer et al. [25] on the basis of 240 bariatric patients with obesity, of whom 80% could discontinue insulin and oral hypoglycemic drugs postoperatively.

In our study of 73 patients with T2DM and morbid obesity, regression of T2DM was observed in 51 cases (69.8%) as early as during hospitalization after LRYGB. In 14 additional patients (19.1%), glycemia and HBA1c were stabilized within 12 weeks after the surgery. The criteria for diagnosing resolution of T2DM, defined as HbA1c level <6% and glucose fasting level <100 mg/dl, were met by as many as 88.9% of the patients. In the remaining eight patients (11%) there was still the need for antidiabetic medication. Those patients had initially long-standing T2DM with long-lasting insulin therapy over 10 years. Obesity, dyslipidemia, type 2 diabetes, hypertension, and other components of metabolic syndrome actively correlate with development of nonalcoholic fatty liver disease (NAFLD) and hepatic insulin resistance and inadequate diabetes resolution after RYGB [26].

An interesting comparison has been undertaken by Geloneze et al. [27]. One-hundred eighty patients with T2DM were dichotomized to surgical treatment or conventional treatment (control group, CG). At 24 weeks after surgery, the surgical patients achieved greater reductions in fasting glucose level (14 vs. 7%) and HbA1c [from 8.78 to 7.84 in RYGB (*p* < 0.01) and from 8.93 to 8.71 in CG (*p* < 0.05 between groups)] and reductions of average daily insulin requirement (93 vs. 29%, *p* < 0.01). Ten patients in RYGB group stopped insulin but they remained on oral hypoglycemic medications. As a conclusion of this article, duodenal-jejunal exclusion was an effective treatment for nonobese T2DM subjects and it was superior to standard care in achieving better glycemic control along with reduction in insulin requirements.

In a meta-analysis by Fried et al. [28], the criteria of T2DM resolution were relatively strict (fasting glycemia <99 mg/dl, HbA1c <6%, plus complete lack of any T2DM conservative treatment). Positive results were achieved in 81.8% of cases. RYGB and BPD were considered the most effective procedures.

The statement presented at the 2nd World Congress on Interventional Therapies for Type 2 Diabetes in March 2011 in New York [9] emphasized that bariatric surgery could be considered an acceptable treatment option for patients with T2DM who have a BMI > 35 kg/m^2^ and for those with a BMI between 30 and 35 kg/m^2^ in whom disease cannot be controlled adequately by medication, especially if they have other major cardiovascular disease risk factors.

The impact of biliopancreatic limb length on the resolution of diabetes that has been presented in our study is still unclear and needs further investigations in larger groups of patients. It is an additional observation noticed during the study, which is still being continued. That is why it is so difficult to finally confirm whether a longer biliopancreatic limb provides a higher rate of resolution of T2DM. In the available literature there is also no definitive answer to this question [29, 30].

It should be underlined that the current study is preliminary and carries a number of potential shortcomings that may result in bias. First, it is observational. Second, the patient group is relatively small. Nevertheless, we believe that the study includes important information about the place metabolic surgery has in the treatment of T2DM.

In summary, the results published worldwide seem promising and they encourage the use of bariatric procedures with the exclusion of the initial part of the small intestine in patients with diabetes and morbid obesity as well as in those overweight or even with normal body weight [31, 32]. Our experience arising from using RYGB in diabetic patients with BMI > 35 kg/m^2^ supports a similarly optimistic view of the future of metabolic surgery. Both reduction of the biochemical measures of T2DM (glycemia and HbA1c) as well as no hypoglycemic drugs needed more than 2 years after the surgery add to the international enthusiasm about this alternative treatment of T2DM. Our preliminary unpublished data on T2DM patients with BMI < 35 kg/m^2^ suggest that similarly impressive T2DM resolution rates can be achieved in this group of patients. It supports the need for further studies in T2DM patients with BMI < 35 kg/m^2^.

## Conclusion

The ultimate evaluation of using bariatric surgery for the treatment of type 2 diabetes is still lacking and requires several years of meticulous clinical studies. Despite that, considering the high cost of life-long conservative therapy of T2DM and its complications, the severe impact it has on the quality of life, and the serious consequences of the disease, surgical metabolic intervention may become the most reasonable solution in many cases.
